# Enhancing pancreatic cancer staging with large language models: the role of retrieval-augmented generation

**DOI:** 10.1007/s12194-026-01026-0

**Published:** 2026-03-05

**Authors:** Hisashi Johno, Yuki Johno, Akitomo Amakawa, Junichi Sato, Ryota Tozuka, Atsushi Komaba, Hiroaki Watanabe, Hiroki Watanabe, Chihiro Goto, Hiroyuki Morisaka, Hiroshi Onishi, Kazunori Nakamoto

**Affiliations:** 1 Department of Radiology, Faculty of Medicine, University of Yamanashi, 1110 Shimokato, Chuo, Yamanashi Japan; 2 Department of Gastroenterology and Hepatology, Faculty of Medicine, University of Yamanashi, Chuo, Yamanashi Japan; 3 Department of Internal Medicine, Kyonan Medical Center Fujikawa Hospital, Fujikawa, Yamanashi Japan; 4 Center for Medical Education and Sciences, Faculty of Medicine, University of Yamanashi, Chuo, Yamanashi Japan

**Keywords:** Large language model (LLM), Retrieval-augmented generation (RAG), Reliable external knowledge (REK), NotebookLM, Gemini 2.0 Flash, Pancreatic cancer staging

## Abstract

Retrieval-augmented generation (RAG) is an emerging technique that enhances large language models (LLMs) by retrieving relevant information from reliable external knowledge (REK). Despite its success in various language processing tasks, the clinical application of RAG in radiology is still novel. To evaluate the utility of RAG in radiological staging tasks, we compared the performance of NotebookLM, a RAG-equipped LLM (RAG-LLM), with its internal model, Gemini 2.0 Flash. Using Japan’s current pancreatic cancer staging guideline as REK, we compared three LLM settings—(1) NotebookLM with REK (REK+/RAG+), (2) Gemini 2.0 Flash with REK (REK+/RAG−), and (3) Gemini 2.0 Flash without REK (REK−/RAG−)—in staging 100 fictional pancreatic cancer cases based on CT findings. Staging tasks included assessment of TNM classification, local invasion factors, and resectability classification. The REK+/RAG+ group achieved 70% staging accuracy, outperforming REK+/RAG− (38%) and REK−/RAG− (35%). For TNM classification, REK+/RAG+ reached 80% accuracy, compared to 55% and 50% in REK+/RAG − and REK−/RAG−, respectively. NotebookLM also presented retrieved REK excerpts as rationale, with a retrieval accuracy of 92%. These results suggest that the RAG system implemented in NotebookLM improves LLM staging performance by enabling access to up-to-date medical guidelines. Furthermore, its ability to retrieve and present source evidence enhances transparency and allows users to verify the reliability of model outputs. This study highlights the potential of a specific RAG system (NotebookLM) to support pancreatic cancer staging by combining clinical language interpretation with direct reference to authoritative guidelines, in an idealized and controlled proof-of-concept experimental setting.

**Supplementary Information:**

The online version contains supplementary material available at 10.1007/s12194-026-01026-0.

## Introduction

Large language models (LLMs) have recently attracted attention in radiology, particularly for their potential to assist in image diagnosis and classification. However, their clinical application remains challenging, partly due to the risk of generating incorrect responses or providing answers unsupported by reliable evidence [1]. One strategy to address these challenges is retrieval-augmented generation (RAG), which enhances the accuracy and reliability of LLM-generated responses by retrieving relevant information from reliable external knowledge (REK) and incorporating it into the model’s prompt [2, 3]. While numerous applications of LLMs have been reported in the field of radiology, studies specifically focusing on LLMs with RAG (RAG-LLMs) remain scarce [4, 5]. These include studies demonstrating the superiority of RAG-LLMs over standard LLMs in radiology board examination performance [6, 7], in adhering to appropriateness criteria for radiologic imaging decisions [8], and in image interpretation tasks [9–11].

Here, we provide a more detailed overview of prior studies related to RAG-LLMs for image interpretation, which is also the focus of our present work. For example, Rau et al. reported that a RAG-equipped chatbot based on GPT-4 generated more appropriate differential diagnoses in gastrointestinal radiology than a generic GPT-4 chatbot, by retrieving 96 domain-specific documents [9]. Choi et al. applied a RAG-LLM to PET imaging reports, demonstrating its ability to reference similar prior cases, with results indicating that it outperformed a non-RAG LLM in terms of the appropriateness of the suggested differential diagnoses [10]. Our previous study also evaluated the utility of NotebookLM, a RAG-LLM developed by Google, for lung cancer staging, demonstrating that the RAG-LLM improved classification accuracy compared to a non-RAG model [11].

Nevertheless, these prior studies did not allow for a pure comparison of the presence or absence of RAG techniques. In the studies by Rau et al. and Choi et al. [9, 10], the external documents used for RAG were not provided to the non-RAG LLMs, making it impossible to isolate the effect of RAG from that of having mere access to external information. In our previous study [11], the non-RAG LLM was indeed given the full external content as part of the prompt; however, NotebookLM (a RAG-LLM based on Gemini 1.5 Pro at the time) was compared with GPT-4o, so the observed differences could not be attributed solely to RAG but were confounded by performance differences between the underlying base language models (Gemini 1.5 Pro and GPT-4o).

Based on these considerations, we designed the present study to isolate the impact of the RAG system implemented in NotebookLM under controlled conditions. Specifically we employed identical base language models for both RAG and non-RAG settings. To ensure a fair comparison, the external information retrieved and utilized by the RAG-LLM was also provided to the non-RAG LLM via direct prompt injection. This experimental design allowed us to attribute observed performance differences to the presence or absence of this specific RAG system, eliminating confounding effects related to base language performance or information access. Furthermore, rather than repeating the application to lung cancer staging, we applied the same methodology to pancreatic cancer, which is the 12th most common cancer worldwide and the sixth leading cause of cancer-related mortality, with an increasing incidence worldwide [12]. Staging tasks in pancreatic cancer are particularly complex, especially in Japan, where the classification system defines local invasion factors, T classification, and resectability classification based on overlapping yet distinct criteria, making staging decisions especially difficult [13].

The purpose of this study was threefold. First, we aimed to isolate the impact of the RAG system implemented in NotebookLM on LLM performance in staging tasks. Second, we sought to assess its performance in complex pancreatic cancer staging tasks. Third, we aimed to explore how the guideline retrieval system could be used for pancreatic cancer staging in an idealized, controlled, proof-of-concept experimental setting. To this end, we evaluated whether NotebookLM is also useful for staging pancreatic cancer as a different type of cancer. Additionally, by comparing it with Gemini 2.0 Flash, the LLM integrated into NotebookLM during the study period (January 2025), we aimed to assess the impact of the RAG system implemented in NotebookLM while minimizing the influence of differences in the underlying language model.

## Materials and methods

**Fig. 1:**
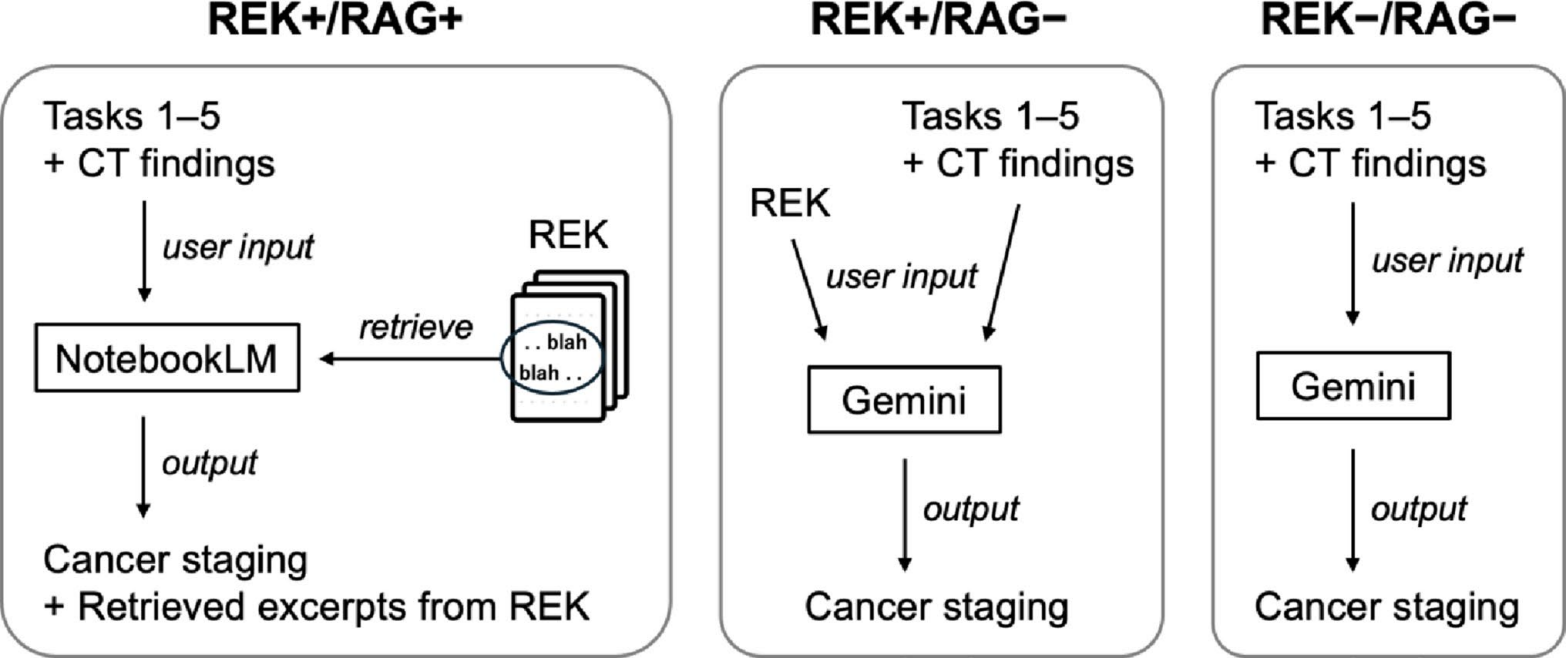
An overview of the experimental process. Radiologists from our team generated CT findings for 100 fictional pancreatic cancer patients. NotebookLM with REK (REK+/RAG+), Gemini 2.0 Flash with REK (REK+/RAG−), and Gemini 2.0 Flash without REK (REK−/RAG−) conducted cancer staging based on the CT findings in response to Tasks 1–5 (see Table 2). *REK* = reliable external knowledge, *RAG* = retrieval-augmented generation

An overview of the experimental process is schematically summarized in Fig. 1. As shown in this figure, we compared the accuracy of cancer staging across three groups: REK+/RAG+ (NotebookLM with REK), REK+/RAG− (Gemini 2.0 Flash with REK), and REK−/RAG− (Gemini 2.0 Flash without REK). Among these, the REK+/RAG+ group represents our proposed method, while the other two groups served for comparison. In the REK+/RAG+ group, the REK was uploaded to the NotebookLM web system, which automatically retrieved only the excerpts relevant to the user input for staging and presented them together with the model’s classification outputs. In the REK+/RAG− group, the entire REK text was manually provided to the LLM without retrieval, so the model processed the full document directly. In the REK−/RAG− group, no REK was given to the LLM, and staging relied on the CT findings together with the model’s pretraining knowledge. At the time of the experiment, the LLM integrated into NotebookLM was Gemini 2.0 Flash; thus, the underlying language model was the same across all three groups. For Gemini 2.0 Flash, the temperature parameter was set to zero to promote output consistency.

### Data preparation

Two radiologists (in their 6th and 11th years of clinical practice, respectively; the latter is board-certified) from our team generated CT findings for 100 fictional pancreatic cancer patients, along with staging components (TNM classification, local invasion factors, and resectability classification) based on the latest pancreatic cancer staging guidelines in Japan—the eighth edition of the Japanese classification of pancreatic carcinoma [13]. The CT findings and staging components were subsequently reviewed and confirmed by four additional radiologists (two in their 7th year, one board-certified in the 12th year, and one board-certified abdominal imaging specialist in the 21st year of clinical practice) and one gastroenterologist (with 8 years of clinical practice, board-certified). A breakdown of the staging components for the 100 fictional pancreatic cancer patients is provided in Table 1. The number of cases is comparable to prior clinical studies [14, 15], but at this sample size, replicating real-world distributions inevitably leads to the absence of certain staging components. To address this, we designed the dataset to reflect the general imbalance observed in clinical practice—particularly in T-factors and local invasion patterns—while also ensuring coverage of the full range of categories. Specifically, we intentionally included a small number of rare cases so that no staging component was entirely absent. Six radiologists confirmed that the distribution reasonably reflects clinical practice. All the CT findings with staging components are available in Supplementary file 1. Below is an example from the dataset, presenting the first of the 100 cases:

*Case 1 CT findings:* *A nodular pancreatic cancer measuring 20 mm is observed in the body of the pancreas.* *No local invasion factors are noted. Lymph node metastases are identified in two nodes at station 10 and two nodes at station 11d. No other metastases are observed.*

*Case 1 staging components: T factor: T1c; N factor: N1b; M factor: M0; Local invasion factors: CH0, DU0, S0, RP0, PV0, A0, PL0, OO0; Resectability classification: R.*

**Table 1:** Breakdown of staging components for the 100 fictional pancreatic cancer patients

T factor	T0	Tis	T1a	T1b	T1c	T2	T3	T4
Number of patients	2	8	4	6	8	4	57	11

N factor	N0	N1a	N1b					
Number of patients	58	22	20					

M factor	M0	M1						
Number of patients	67	33						

Local invasion factors	CH1	DU1	S1	RP1	PV1	A1	PL1	OO1
Number of patients	7	4	62	64	47	34	7	16

Resectability classification	R	BR	UR					
Number of patients	42	16	42					

### Preparation of REK and user input

We attempted to use an open-access paper [13], which summarizes the current pancreatic cancer staging guidelines in Japan, as REK for NotebookLM and Gemini 2.0 Flash. However, since the full text of the paper (5,418 words, covering Chaps. 1 to 8, including figure captions and tables) could not be entered into the prompt field at once, we adopted REK consisting of 4,376 words from the paper (Chaps. 1 to 6), omitting the final two chapters that were not relevant to staging.

To enable the LLM to perform pancreatic cancer staging, we provided Tasks 1–5, shown in Table 2, as user input in the prompt field, followed by the CT findings for each case. In the REK+/RAG+ group, we uploaded the REK to the NotebookLM web system for RAG processing. In the REK+/RAG− group, we manually entered the REK into the prompt field before providing Tasks 1–5. In the REK−/RAG− group, we entered a prompt instructing adherence to the Japanese Classification of Pancreatic Carcinoma, Eighth Edition by the Japan Pancreas Society before providing Tasks 1–5. To assess potential variability in staging results, identical inputs were submitted twice for each of the 100 cases under each LLM setting across independent runs, and the resulting staging classifications were consistent.

**Table 2:** User input for LLMs to perform pancreatic cancer staging

Task 1	Diagnose the local invasion factors of pancreatic cancer (CH, DU, S, RP, PV, A, PL, OO) and respond in the format: e.g., “CH0, DU1, S1, RP1, PV0, A0, PL0, OO1”.
Task 2	Based on the answer to Task 1, determine the T classification of pancreatic cancer (T0, Tis, T1a, T1b, T1c, T2, T3, T4).
Task 3	Determine the N classification of pancreatic cancer (N0, N1a, N1b) based on the defined criteria for regional lymph nodes. Note that Metastasis to non-regional lymph nodes is classified under M classification, not N classification.
Task 4	Determine the M classification of pancreatic cancer (M0, M1).
Task 5	Based on the answer to Task 4, determine the resectability classification of pancreatic cancer (R, BR, UR). Respond with BR if the classification is BR-PV or BR-A, and UR if it is UR-LA or UR-M. If both BR and UR apply, respond with UR.

### Evaluation

For a given case, cancer staging was regarded as correct if all staging components—TNM classification, local invasion factors, and resectability classification—were simultaneously determined correctly. Staging accuracy was then defined as the proportion of correctly staged cases among the 100 examined. Staging accuracy was compared across the three groups: REK+/RAG+, REK+/RAG−, and REK−/RAG−. For staging accuracy, the exact McNemar’s test was applied. Based on the observed concordant and discordant outcomes per case, we tested the null hypothesis that the population proportions of correctly staged cases were equal between groups, and calculated the corresponding *p*-values. Specifically, pairwise comparisons were performed between the REK+/RAG + and REK+/RAG− groups, between the REK+/RAG − and REK−/RAG− groups, and between the REK+/RAG + and REK−/RAG− groups. Additionally, for each staging component (T, N, M factors, and resectability), accuracy was defined as the proportion of cases (out of the 100 examined) in which the component was correctly classified. The TNM classification was considered correct only when all T, N, and M factors were accurately classified. For local invasion factors, a case was considered correct only when all eight factors (CH, DU, S, RP, PV, A, PL, and OO) were simultaneously classified correctly. For both the TNM classification and local invasion factors, accuracy was defined as the proportion of correctly classified cases among the 100 examined.

In the REK+/RAG+ group, retrieved excerpts from REK via NotebookLM were available for reference. Therefore, we examined these excerpts for each case and evaluated retrieval accuracy. Retrieval was considered accurate if the excerpts contained sufficient information to correctly classify all of the staging components. This definition is based on the *context recall* measure in the RAGAS framework [16–18], a recently developed suite of metrics for evaluation of RAG. Specifically, let $\:C$ denote the set of all chunks (i.e., smaller text units) obtained from REK by NotebookLM. For each case $\:i=1,\:\dots\:,\:100$, let $\:{C}_{i}\subset\:C$ be the retrieved set, let $\:{\{A}_{1}^{i},\:\dots\:,\:{A}_{k}^{i}\}$ be the set of claims of the correct answer, and let

$$\:{f}_{i}\left({A}_{j}^{i}\right)=\left\{\begin{array}{c}1\:\:\:\:\mathrm{if}\:{A}_{j}^{i}\:\text{can be attributed to}\:{C}_{i}\\\!\!\!\!\!\!\!0\:\:\:\:\mathrm{otherwise}\:\:\:\:\:\:\:\:\:\:\:\:\:\:\:\:\:\:\:\:\:\:\:\:\:\:\:\:\:\:\:\:\end{array}\:\:\:\:\:\:\:\:\left(j=1,\dots\:,k\right).\right.$$

Then, the context recall for case $\:i$ is defined as

$$ \:{\mathrm{CR}}_{i} = \frac{1}{k}\sum\limits_{{j = 1}}^{k} {f_{i} \left( {A_{j}^{i} } \right)} $$

Since $\:\mathrm{C}{\mathrm{R}}_{i}<1$ indicates insufficient retrieval, we applied the floor function $\left\lfloor \cdot \right\rfloor$ to obtain a stricter, more conservative evaluation and defined the retrieval accuracy for the overall 100 cases as

$$ \:{\mathrm{RA}} = \frac{1}{{100}}\sum\limits_{{i = 1}}^{{100}} {\left\lfloor {{\mathrm{CR}}_{i} } \right\rfloor } $$

        

For each case $\:i$, the definition of $\:{f}_{i}$ was independently reviewed by one radiologist (with 11 years of clinical practice, board-certified), one gastroenterologist (with 8 years of clinical practice, board-certified), and one engineer with medical training, to ensure consistency in claim attribution. Occasional discrepancies due to minor misunderstandings were observed, but all were resolved through mutual review, resulting in inter-rater agreement on the final definition of every $\:{f}_{i}$.

The LLM’s answers for each case in the three groups (REK+/RAG+, REK+/RAG−, and REK−/RAG−), along with the retrieved excerpts in the REK+/RAG+ group, are provided in Supplementary file 2 to enable transparent verification by readers. Additionally, the case-wise accuracy of each staging component across the three groups is presented in Supplementary file 3, while the case-wise retrieval accuracy in the REK+/RAG+ group is provided in Supplementary file 4. Furthermore, Supplementary file 5 summarizes, for each model, the number of correctly classified cases for every staging component.

## Results

**Fig. 2:**
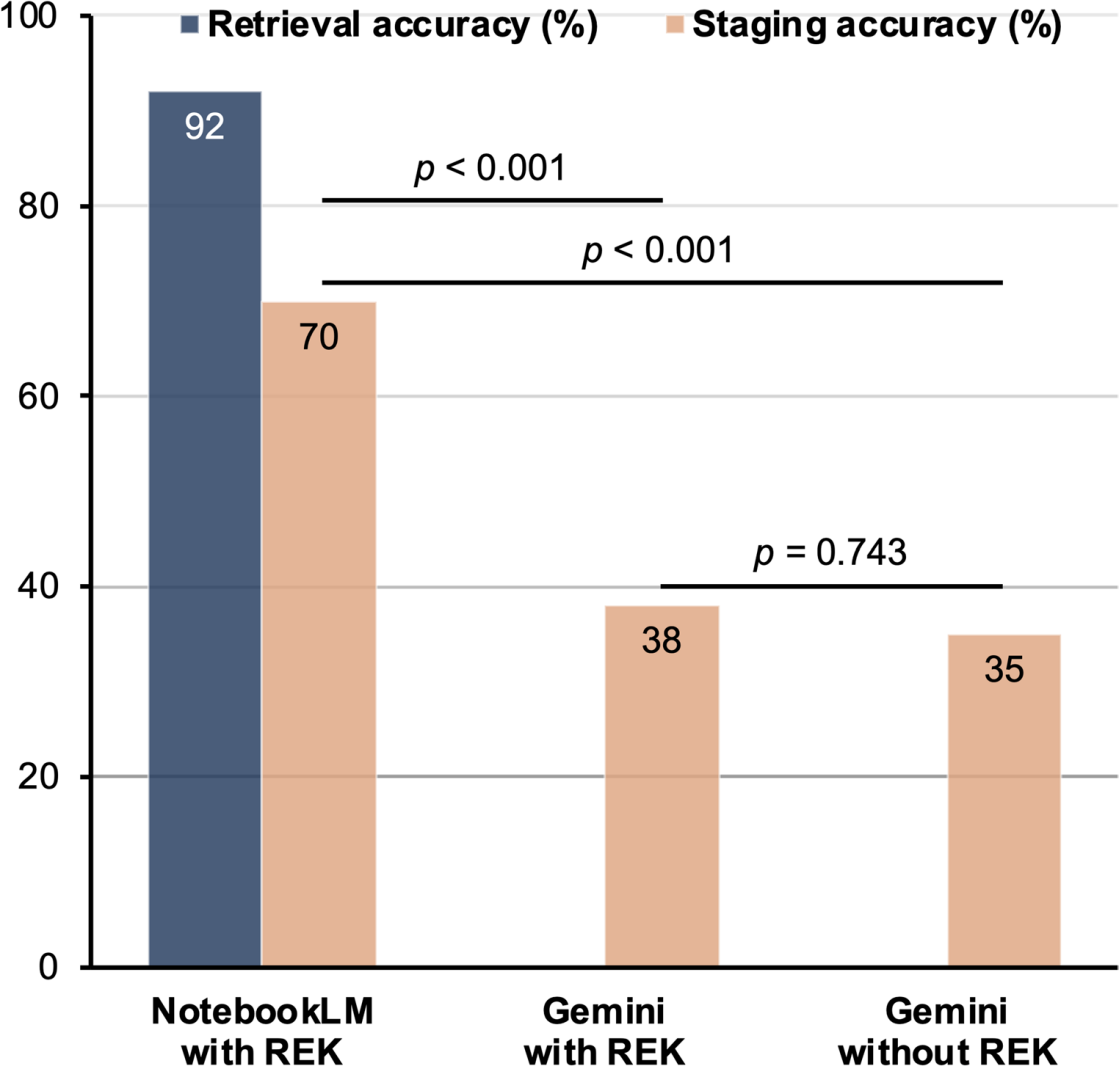
Staging performance of NotebookLM with REK (REK+/RAG+), Gemini 2.0 Flash with REK (REK+/RAG−), and Gemini 2.0 Flash without REK (REK−/RAG−) in the experiment using 100 fictional pancreatic cancer cases. Staging was considered accurate if all the staging components—TNM classification, local invasion factors, and resectability classification—were correctly determined. For NotebookLM (REK+/RAG+), retrieval accuracy was also evaluated. Retrieval was considered accurate if the retrieved excerpts from REK contained sufficient information to enable accurate cancer staging (see Materials and Methods for the explicit definition). Pairwise comparisons of staging accuracy using the exact McNemar’s test yielded *p* < 0.001 for NotebookLM with REK (REK+/RAG+) versus Gemini 2.0 Flash with REK (REK+/RAG−), *p* = 0.743 for Gemini 2.0 Flash with REK (REK+/RAG−), versus Gemini 2.0 Flash without REK (REK−/RAG−), and *p* < 0.001 for NotebookLM with REK (REK+/RAG+) versus Gemini 2.0 Flash without REK (REK−/RAG−). *REK* = reliable external knowledge

In the experiment using 100 fictional pancreatic cancer cases, NotebookLM with REK (REK+/RAG+) achieved a staging accuracy of 70%, whereas Gemini 2.0 Flash with REK (REK+/RAG−) and without REK (REK−/RAG−) showed lower accuracies of 38% and 35%, respectively (Fig. 2). Pairwise comparisons of staging accuracy using the exact McNemar’s test showed a difference of 0.32 (95% confidence interval: [0.17, 0.45]) between NotebookLM with REK (REK+/RAG+) and Gemini 2.0 Flash with REK (REK+/RAG−) (*p* < 0.001). The difference between Gemini 2.0 Flash with REK (REK+/RAG−) and Gemini 2.0 Flash without REK (REK−/RAG−) was 0.03 (95% confidence interval: [− 0.13, 0.19]) (*p* = 0.743). The difference between NotebookLM with REK (REK+/RAG+) and Gemini 2.0 Flash without REK (REK−/RAG−) was 0.35 (95% confidence interval: [0.19, 0.49]) (*p* < 0.001).

**Fig. 3:**
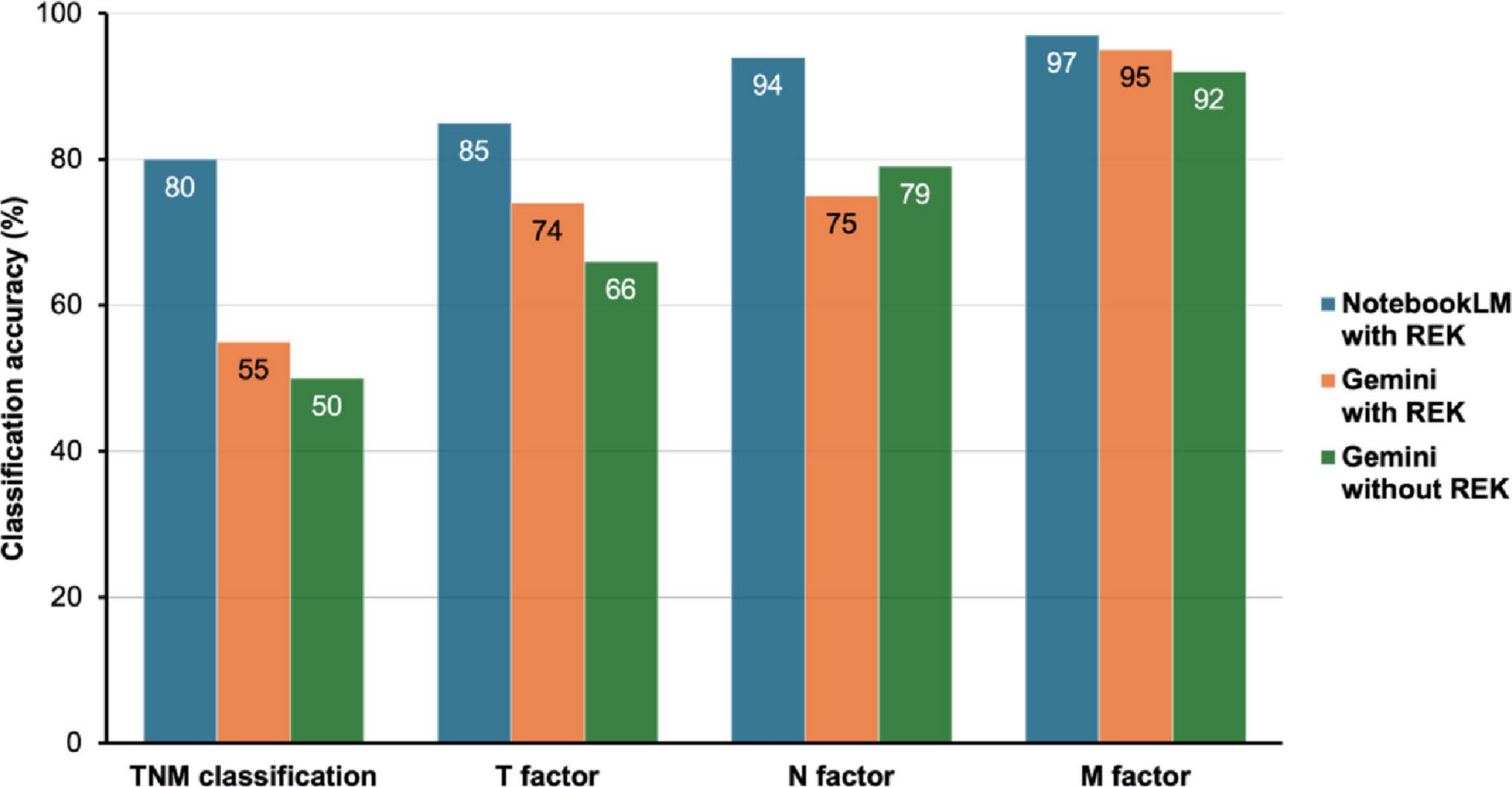
TNM classification performance of NotebookLM with REK (REK+/RAG+), Gemini 2.0 Flash with REK (REK+/RAG−), and Gemini 2.0 Flash without REK (REK−/RAG−) in the experiment using 100 fictional pancreatic cancer cases. The TNM classification was deemed correct only if all T, N, and M factors were accurately identified. Additionally, the classification accuracy for each T, N, and M factor was compared across the three groups. *REK* = reliable external knowledge

**Fig. 4:**
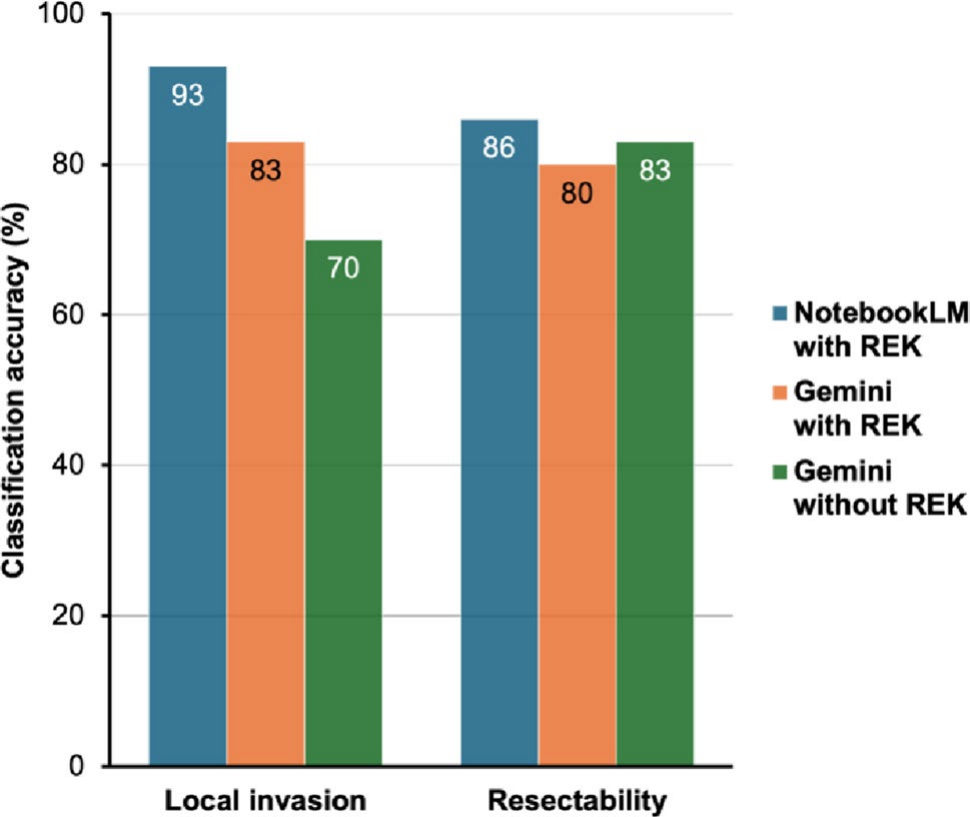
The performance of NotebookLM with REK (REK+/RAG+), Gemini 2.0 Flash with REK (REK+/RAG−), and Gemini 2.0 Flash without REK (REK−/RAG−) in determining local invasion factors and resectability classification in the experiment using 100 fictional pancreatic cancer cases. *REK* = reliable external knowledge

For TNM classification, NotebookLM with REK (REK+/RAG+) achieved an accuracy of 80%, outperforming Gemini 2.0 Flash with REK (REK+/RAG−, 55%) and without REK (REK−/RAG−, 50%), with a notable advantage in T and N factors (Fig. 3). A similar trend was seen in the classification accuracy of local invasion factors; however, the advantage of NotebookLM (REK+/RAG+) in resectability classification was not distinct (Fig. 4). The subgroup summaries (Supplementary file 5) indicate that even NotebookLM with REK (REK+/RAG+), the best-performing model overall, showed relatively lower accuracy particularly in distinguishing T3 from other categories in the T classification and in separating R, BR, and UR in the resectability classification. Among the misclassified T3 cases by NotebookLM with REK (REK+/RAG+), 12 were overstaged as T4, whereas 2 were understaged as T2 or lower. Regarding resectability, among the misclassified R cases, 1 was overstaged as BR and 7 as UR. All three misclassified BR cases were overstaged as UR. In contrast, among the misclassified UR cases, 2 were understaged as R and 1 as BR.

**Fig. 5:**
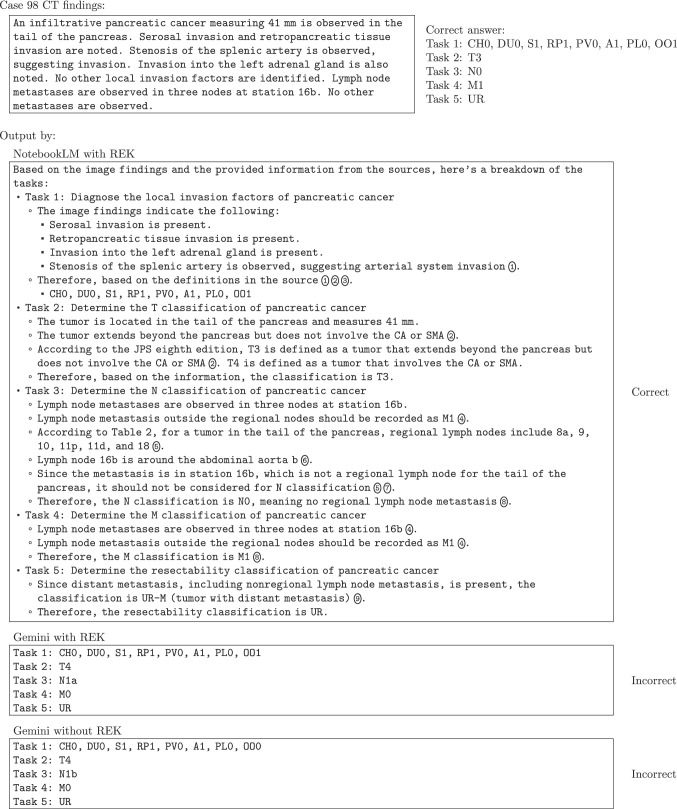
A representative result from the pancreatic cancer staging experiment (Case 98). In this case, both staging and retrieval by NotebookLM (REK+/RAG+) were correct, whereas staging by Gemini 2.0 Flash with REK (REK+/RAG−) and without REK (REK−/RAG−) was incorrect. A subset of the REK excerpts retrieved by NotebookLM (REK+/RAG+) is available in Supplementary file 6, while the full set (from ① to ⑨) can be found in Supplementary file 2. *REK* = reliable external knowledge

**Fig. 6:**
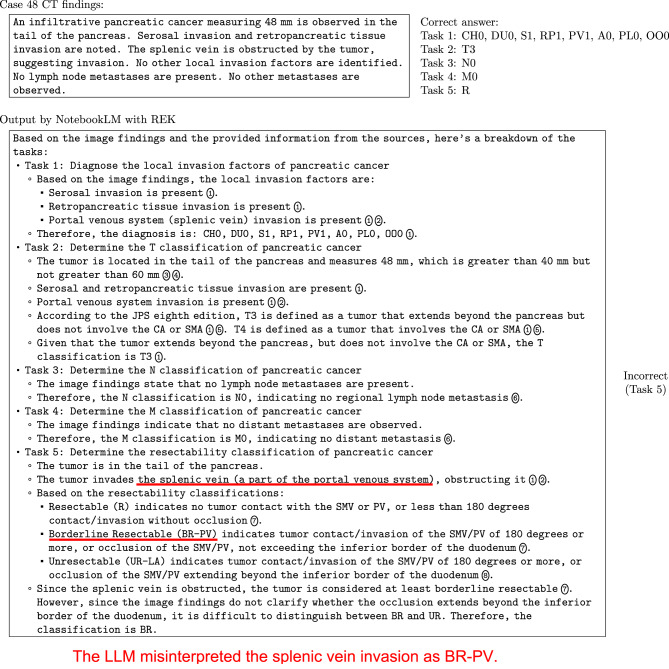
A representative result from the pancreatic cancer staging experiment (Case 48). In this case, although NotebookLM (REK+/RAG+) appropriately retrieved REK excerpts, the LLM misinterpreted the information, leading to an incorrect staging. A subset of the REK excerpts retrieved by NotebookLM (REK+/RAG+) is available in Supplementary file 7, while the full set (from ① to ⑧) can be found in Supplementary file 2. *REK* = reliable external knowledge, *LLM* = large language model

Unlike Gemini 2.0 Flash with REK (REK+/RAG−) and without REK (REK−/RAG−), NotebookLM (REK+/RAG+) presented retrieved excerpts from REK as the basis for its classifications, achieving a retrieval accuracy of 92% (Fig. 2). For example, Fig. 5 shows the experimental results for Case 98. While Gemini 2.0 Flash with REK (REK+/RAG−) and without REK (REK−/RAG−) output only the classification results, which were incorrect, NotebookLM (REK+/RAG+) explicitly provided the retrieved REK excerpts as the basis for its answers and correctly solved all tasks (a subset of the retrieved excerpts is shown in Supplementary file 6). In this case, Gemini 2.0 Flash with REK (REK+/RAG−) misclassified T, N, and M classifications (Tasks 2–4), while Gemini 2.0 Flash without REK (REK−/RAG−) misclassified all of these factors plus the local invasion factor OO (Task 1). Since the non-RAG settings did not provide any supporting rationale based on the REK, it was difficult to determine the cause of errors. In contrast, while NotebookLM (REK+/RAG+) did not always produce correct answers, it consistently provided explanatory context grounded in the REK, enabling easier identification of the source of error by examining the corresponding REK excerpts. For example, Fig. 6 shows the results of Case 48, where NotebookLM (REK+/RAG+) successfully retrieved relevant excerpts but still produced an incorrect staging output. Although sufficient information for correct resectability classification was retrieved from REK (Supplementary file 7), the model mistakenly interpreted splenic vein invasion as BR-PV and misclassified Task 5 as BR instead of the correct label R, despite correctly answering Tasks 1–4 (Fig. 6). There were a few cases (eight in total) in which retrieval from REK was inaccurate. For example, in Case 59, the retrieved excerpts lacked information on “Resectable: R,” which was necessary for accurate resectability classification (Supplementary file 2). Among the eight retrieval failure cases, five involved missing information for resectability classification, two for T classification, and one for local invasion factors. No staging category was correctly classified when a retrieval failure occurred. Consequently, retrieval failures accounted for a minority of misclassifications (resectability: 5/14, T category: 2/15, local invasion: 1/7), whereas most errors occurred despite sufficient retrieval, indicating generation-related failures (see Figs. 3 and 4 for reference).

## Discussion

In this study, we demonstrated that the RAG-LLM framework (NotebookLM; REK+/RAG+) achieved superior accuracy in pancreatic cancer staging compared with the baseline LLM (Gemini 2.0 Flash) without RAG (REK+/RAG−), even though both models were provided with the same REK. Specifically, NotebookLM (REK+/RAG+) achieved a staging accuracy of 70% and a TNM classification accuracy of 80%, whereas Gemini 2.0 Flash without RAG (REK+/RAG−) achieved 38% and 55%, respectively. These results isolate the contribution of the RAG system implemented in NotebookLM. Because real-world radiologic findings may include ambiguity or incomplete information that could adversely affect LLM classification accuracy, these performance values should be interpreted as upper-bound estimates obtained under idealized and controlled conditions with complete and unambiguous fictional cases, rather than as performance in real-world clinical settings. By extending our previous work on lung cancer staging to pancreatic cancer, we further showed that the benefit of RAG is not limited to a single cancer type. Notably, the RAG-LLM was able to provide retrieved REK excerpts as explicit evidence for staging. Our findings align with and extend previous reports on the use of LLMs for pancreatic cancer staging. Bhayana et al. evaluated GPT-3.5 and GPT-4 for generating synoptic radiology reports and assessing tumor resectability, and reported that GPT-4 achieved near-perfect synoptic reports while chain-of-thought prompting improved resectability classification accuracy; however, clinically significant misinterpretations persisted, highlighting the need for physician supervision [14]. Similarly, Suzuki et al. assessed GPT-4 for TNM classification using Japanese radiology reports and found its performance inadequate for clinical standards [15]. Consistent with these observations, in our experiment, both conditions without RAG (REK+/RAG − and REK−/RAG−) showed poor performance, whereas the RAG-LLM (REK+/RAG+) demonstrated clear improvements (Figs. 2, 3 and 4). Taken together, we extend prior work—which reported limited accuracy with standalone LLMs in pancreatic cancer staging—by demonstrating that the RAG system implemented in NotebookLM can effectively improve staging accuracy.

Although NotebookLM exhibited superior performance in cancer staging, the majority of classification errors persisted even when relevant excerpts from REK were appropriately retrieved, as the LLM misinterpreted the retrieved information at the generation stage. Misinterpretations (or hallucinations) remain a serious concern in the field of LLM research, and no complete solution has been found [19]. Therefore, even a highly accurate LLM poses risks if used in medical practice without physician oversight, reinforcing the need to limit its role to a supplementary tool rather than a fully autonomous one. For guideline-based tasks like cancer staging, LLM-generated classifications without reliable supporting evidence are unlikely to reduce physicians’ workload, as they would still need to consult the guidelines just as they would without LLM assistance. However, this study demonstrated that the RAG-LLM, NotebookLM, can explicitly provide retrieved REK excerpts as evidence for staging (see Figs. 5 and 6, as well as Supplementary files 6 and 7, for examples) with relatively high accuracy (Fig. 2), suggesting the potential to facilitate physician verification of staging decisions and to help identify hallucinated outputs. While this implication is indirectly supported by our results, future studies should directly evaluate whether the provision of partial REK through RAG technology effectively improves efficiency and reduces physicians’ burden. In addition, future studies should also examine whether the use of RAG-LLMs as a second opinion improves physicians’ diagnostic accuracy. As an important consideration, the usefulness of this approach depends on the reliability of the REK itself. In our study, the REK was based on an official guideline issued by the Japan Pancreas Society. For clinical applications, users must ensure that only validated and trustworthy documents are used as REK.

The remaining limitations and future perspectives of this study are as follows. First, unlike in actual clinical settings, we evaluated the LLM’s staging accuracy using fictional cancer CT findings and Japan’s pancreatic cancer staging guidelines in English rather than Japanese. In addition, as noted earlier, the dataset did not fully replicate real-world distributions across staging components, in part due to the limited sample size. Moreover, the fictional cases were intentionally designed to include clean and unambiguous imaging findings with complete information for staging, which does not fully reflect the ambiguity and incompleteness occasionally encountered in real-world radiology reports. To rigorously assess its applicability in real clinical practice, validation with actual clinical data and a sufficiently large sample size is necessary. In the present results, most misclassified T3 and resectability BR cases were overstaged. Given the potential clinical implications of misclassification direction, including limited surgical options due to overstaging and insufficient surgery due to understaging, further validation using real-world clinical data is warranted. Second, because key RAG parameters in NotebookLM are not publicly available, the contribution of individual retrieval settings cannot be evaluated, the retrieval process cannot be optimized for specific use cases, and the origins of errors within the retrieval-to-generation process cannot be directly traced. Further validation using open systems with explicitly defined RAG configurations is warranted. Third, while this study isolated and evaluated the utility of a particular RAG system (NotebookLM) in pancreatic cancer staging, its generalizability to other RAG configurations, knowledge sources, and cancer types remains to be established. Future research should further investigate its applicability to broader clinical tasks, such as identifying differential diagnoses based on imaging findings.

## Conclusion

This study demonstrated that a RAG-LLM framework improved staging accuracy compared with a baseline LLM without RAG, even though both models were provided with the same REK. This isolates the contribution of the RAG system implemented in NotebookLM. As an extension of our previous work using NotebookLM for lung cancer staging, the present study highlights the potential usefulness of RAG in the complex staging of pancreatic cancer. The utility of RAG-based evidence provision for pancreatic cancer staging is also highlighted in an idealized and controlled proof-of-concept experimental setting.

## Supplementary Information

Below is the link to the electronic supplementary material.

Supplementary Material 1

Supplementary Material 2

Supplementary Material 3

Supplementary Material 4

Supplementary Material 5

Supplementary Material 6

Supplementary Material 7

## Data Availability

Most of the data supporting the findings of this study are included in the article and its Supplementary files. Further details and additional data can be obtained from the corresponding author upon reasonable request.

## References

[1] Keshavarz P, Bagherieh S, Nabipoorashrafi SA, Chalian H, Rahsepar AA, Kim GHJ, et al. ChatGPT in radiology: a systematic review of performance, pitfalls, and future perspectives. Diagn Interv Imaging. 2024;105:251–65. 10.1016/j.diii.2024.04.003. 38679540 10.1016/j.diii.2024.04.003

[2] Lewis P, Perez E, Piktus A, Petroni F, Karpukhin V, Goyal N, et al. Retrieval-augmented generation for knowledge-intensive NLP tasks. Adv Neural Inf Process Syst. 2020;33:9459–74.

[3] Shuster K, Poff S, Chen M, Kiela D, Weston J. Retrieval augmentation reduces hallucination in conversation. Find Assoc Comput Linguist EMNLP. 2021;3784–803. 10.18653/v1/2021.findings-emnlp.320.

[4] Lanzafame LRM, Gulli C, Mazziotti S, Ascenti G, Gaeta M, Vogl TJ, et al. Chatbots in radiology: current applications, limitations and future directions of ChatGPT in medical imaging. Diagnostics (Basel). 2025;15:1635. 10.3390/diagnostics15131635 10.3390/diagnostics15131635 PMC12248470 40647634

[5] Tordjman M, Bolger I, Yuce M, Restrepo F, Liu Z, Dercle L, et al. Large language models in cancer imaging: applications and future perspectives. J Clin Med. 2025;14:3285. 10.3390/jcm14103285 10.3390/jcm14103285 PMC12112367 40429281

[6] Bhayana R, Fawzy A, Deng Y, Bleakney RR, Krishna S. Retrieval-augmented generation for large language models in radiology: another leap forward in board examination performance. Radiology. 2024;313:e241489. 10.1148/radiol.241489. 39377675 10.1148/radiol.241489

[7] Weinert DA, Rauschecker AM. Enhancing large language models with retrieval-augmented generation: a radiology-specific approach. Radiol Artif Intell. 2025;12:e240313. 10.1148/ryai.240313. 10.1148/ryai.240313 40072217

[8] Rau A, Rau S, Zoeller D, Fink A, Tran H, Wilpert C, et al. A context-based chatbot surpasses radiologists and generic ChatGPT in following the ACR appropriateness guidelines. Radiology. 2023;308:e230970. 10.1148/radiol.230970. 37489981 10.1148/radiol.230970

[9] Rau S, Rau A, Nattenmüller J, Fink A, Bamberg F, Reisert M, et al. A retrieval-augmented chatbot based on GPT-4 provides appropriate differential diagnosis in gastrointestinal radiology: a proof of concept study. Eur Radiol Exp. 2024;8:60. 10.1186/s41747-024-00457-x 10.1186/s41747-024-00457-x PMC11098977 38755410

[10] Choi H, Lee D, Kang YK, Suh M. Empowering PET imaging reporting with retrieval-augmented large language models and reading reports database: a pilot single center study. Eur J Nucl Med Mol Imaging. 2025;52:2452–62. 10.1007/s00259-025-07101-9. 39843863 10.1007/s00259-025-07101-9 PMC12119711

[11] Tozuka R, Johno H, Amakawa A, Sato J, Muto M, Seki S, et al. Application of NotebookLM, a large language model with retrieval-augmented generation, for lung cancer staging. Jpn J Radiol. 2025;43:706–12. 10.1007/s11604-024-01705-1. 39585559 10.1007/s11604-024-01705-1

[12] Yu W, Zhou D, Meng F, Wang J, Wang B, Qiang J, et al. The global, regional burden of pancreatic cancer and its attributable risk factors from 1990 to 2021. BMC Cancer. 2025;25:186. 10.1186/s12885-025-13471-y. 39891086 10.1186/s12885-025-13471-y PMC11786447

[13] Ishida M, Fujii T, Kishiwada M, Shibuya K, Satoi S, Ueno M, et al. Japanese classification of pancreatic carcinoma by the Japan Pancreas Society: eighth edition. J Hepatobiliary Pancreat Sci. 2024;31:755–68. 10.1002/jhbp.12056. 39074998 10.1002/jhbp.12056 PMC11589393

[14] Bhayana R, Nanda B, Dehkharghanian T, Deng Y, Bhambra N, Elias G, et al. Large language models for automated synoptic reports and resectability categorization in pancreatic cancer. Radiology. 2024;311:e233117. 10.1148/radiol.233117. 38888478 10.1148/radiol.233117

[15] Suzuki K, Yamada H, Yamazaki H, Honda G, Sakai S. Preliminary assessment of TNM classification performance for pancreatic cancer in Japanese radiology reports using GPT-4. Jpn J Radiol. 2025;43:51–5. 10.1007/s11604-024-01643-y 10.1007/s11604-024-01643-y PMC11717849 39162781

[16] Es S, James J, Espinosa-Anke L, Schockaert S. RAGAS: automated evaluation of retrieval augmented generation. In: Aletras N, De Clercq O, editors. Proc 18th Conf Eur Chapter Assoc Comput Linguist: Syst Demonstrations; 2024 Mar 17–22; St. Julian’s, Malta. Stroudsburg (PA): Assoc Comput Linguist; 2024. p. 150–8. 10.18653/v1/2024.eacl-demo.16

[17] ExplodingGradients. Ragas: supercharge your LLM application evaluations [Internet]. 2024. Available from: https://github.com/explodinggradients/ragas

[18] ExplodingGradients. RAGAS documentation – context recall [Internet]. Version 0.3.2, 2025 Jun 17 [cited 2025 Aug 26]. Available from: https://docs.ragas.io/en/v0.3.2/concepts/metrics/available_metrics/context_recall/

[19] Huang L, Yu W, Ma W, Zhong W, Feng Z, Wang H, et al. A survey on hallucination in large language models: principles, taxonomy, challenges, and open questions. ACM Trans Inf Syst. 2025;43:42. 10.1145/3703155.

