# Supplementary material for: Enhancing pancreatic cancer staging with large language models: the role of retrieval-augmented generation
Source: Radiol Phys Technol. 2026 Mar 5;19(2):593–603. doi: 10.1007/s12194-026-01026-0 (PMC13253659; doi:10.1007/s12194-026-01026-0)
Supplement: Supplementary file 6 — Supplementary Material 6 [file 12194_2026_1026_MOESM6_ESM.docx]

Partial reproduction from the following article:

Ishida M, Fujii T, Kishiwada M, et al. (2024) “Japanese classification of pancreatic carcinoma by the Japan Pancreas Society: Eighth edition”. Journal of Hepato-Biliary-Pancreatic Sciences, Vol. 31, 755–768. <https://doi.org/10.1002/jhbp.12056>

© 2024 The Author(s). Journal of Hepato-Biliary-Pancreatic Sciences published by John Wiley & Sons Australia, Ltd on behalf of the Japanese Society of Hepato-Biliary-Pancreatic Surgery. This is an open access article under the terms of the Creative Commons Attribution License (CC BY 4.0), which permits use, distribution, and reproduction in any medium, provided the original work is properly cited. https://creativecommons.org/licenses/by/4.0/
